# Optimizing Insecticide Resources: Global Trends in Vector Control

**DOI:** 10.1289/ehp.120-a164b

**Published:** 2012-04-01

**Authors:** Tanya Tillett

**Affiliations:** Tanya Tillett, MA, of Durham, NC, is a staff writer/editor for *EHP*. She has been on the *EHP* staff since 2000 and has represented the journal at national and international conferences.

Although insecticides have helped lower worldwide rates of infectious disease, the use of these chemicals must be monitored and controlled to avoid pest resistance and minimize associated risks to human health and the environment. Researchers have conducted a comprehensive assessment of trends in the global use of insecticides for vector-borne disease control over the last decade and found that the potential for overuse of pyrethroids could result in a loss of the effectiveness of long-lasting insecticidal nets (LNs), which have been one of the most successful tools for controlling malaria to date **[*EHP* 120(4):577–582; van den Berg et al.]**.

The research team analyzed data from 125 countries that used organochlorines, organophosphates, carbamates, and pyrethroids for vector control between 2000 and 2009. Application methods were classified as residual spraying (spraying interior and peripheral surfaces of houses), space spraying (spraying exterior spaces), treatment of nets (not including factory manufacturing of LNs, which contain pyrethroids), and larviciding (treating aquatic breeding sites of mosquitoes with insecticides). The team conducted 2 analyses of insecticide use for each country, covering a 10-year and an annual average.

**Figure:**
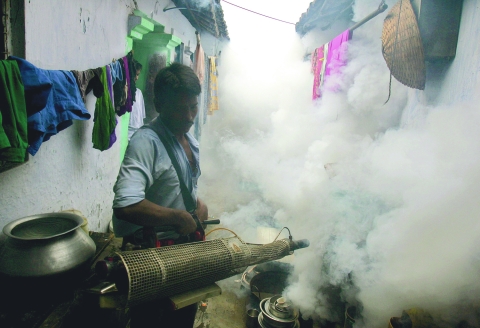
A health officer in Kolkata, India, fumigates to prevent the spread of mosquito-borne diseases in 2005. Reuters/Parth Sanyal PS/VM/PN

The main diseases targeted by insecticide use were malaria, dengue, leishmaniasis, and Chagas disease. The organochlorine DDT was by far the most used insecticide in terms of quantity (71%), with India accounting for most applications (82%) and African countries accounting for the rest. Globally the use of DDT has not changed substantially since the Stockholm Convention on Persistent Organic Pollutants was established in 2004, although use increased sharply in Africa until 2008.

Pyrethroid use in residual and space spraying accounted for the greatest surface or area covered (81%), with most use occurring in the Americas, although an increase occurred in African countries. Global pyrethroid use has increased steadily since 2004, mainly due to increased use for residual spraying, a factor the authors warn could promote resistance among mosquitoes, negatively affecting the long-term effectiveness of treated bed nets. They warn that “in areas where resistance genes have already spread, immediate implementation of resistance management is required to preserve the effectiveness of available tools, including LNs.”

The main limitation of the study is countries’ potential lack of capacity for reporting insecticide use, especially regarding dengue control (which tends to be less structured than malaria control). Even so, the data collected can help guide global strategies on vector control and resistance management.

